# Analysis of ferrite nanoparticles in the flow of ferromagnetic nanofluid

**DOI:** 10.1371/journal.pone.0188460

**Published:** 2018-01-10

**Authors:** Noor Muhammad, Sohail Nadeem, M. T. Mustafa

**Affiliations:** 1 Department of Mathematics, Quaid-I-Azam University 45320, Islamabad 44000, Pakistan; 2 Department of Mathematics, Statistics and Physics, Qatar University, Doha 2713, Qatar; Brandeis University, UNITED STATES

## Abstract

Theoretical analysis has been carried out to establish the heat transport phenomenon of six different ferromagnetic MnZnFe_2_O_4_—C_2_H_6_O_2_ (manganese zinc ferrite-ethylene glycol), NiZnFe_2_O_4_—C_2_H_6_O_2_ (Nickel zinc ferrite-ethylene glycol), Fe_2_O_4_—C_2_H_6_O_2_ (magnetite ferrite-ethylene glycol), NiZnFe_2_O_4_—H_2_O (Nickel zinc ferrite-water), MnZnFe_2_O_4_—H_2_O (manganese zinc ferrite-water), and Fe_2_O_4_—H_2_O (magnetite ferrite-water) nanofluids containing manganese zinc ferrite, Nickel zinc ferrite, and magnetite ferrite nanoparticles dispersed in a base fluid of ethylene glycol and water mixture. The performance of convective heat transfer is elevated in boundary layer flow region via nanoparticles. Magnetic dipole in presence of ferrites nanoparticles plays a vital role in controlling the thermal and momentum boundary layers. In perspective of this, the impacts of magnetic dipole on the nano boundary layer, steady, and laminar flow of incompressible ferromagnetic nanofluids are analyzed in the present study. Flow is caused by linear stretching of the surface. Fourier’s law of heat conduction is used in the evaluation of heat flux. Impacts of emerging parameters on the magneto—thermomechanical coupling are analyzed numerically. Further, it is evident that Newtonian heating has increasing behavior on the rate of heat transfer in the boundary layer. Comparison with available results for specific cases show an excellent agreement.

## 1 Introduction

Heat transfer enhancement in a two-phase fluid flow has been scrutinized for many years. In heat transfer equipment liquids are frequently utilized as heat transporter. Research on nanofluid flow depicts that by adding ferrite nanoparticles in the fluid, the heat transfer coefficient can be enhanced. The resulting increase in the heat transfer, in addition to the possible rise in thermal conductivity, was mainly because of the reduced thickness of the thermal boundary layer. It is very probable that motion of ferrite nanoparticles in the fluid will enhance thermal conductivity and heat transfer. Examples of important uses of heat transfer liquids include hydronic and cooling heating systems in buildings, vehicular and avionics cooling systems in industry, chemical, foods, and other processing plants. In all the mentioned applications, the thermal conductivity of heat transfer liquids play a vital role in the construction of energy-efficient heat transport equipment. It is suggested that nano-meter metallic particles can be suspended in heat transfer fluids such as ethylene glycol, water or engine oil to a new class of fluid with high thermal conductivity, the resulting fluid is termed as nanofluid [1]. Nanofluid displays better quality when compared with fluids containing micrometer-sized particles and conventional heat transfer liquids. Since heat transfer results on the surface of the particle, it is necessary to use nanoparticle with large surface area. Nanoparticles have sufficiently large surface area as compare to micrometer-sized particles, and therefore nanofluids have extensive potential for application [2–10] in heat transfer.

Ferrofluids are colloidal liquids made of ferrimagnetic or ferromagnetic ferrite nanoparticles slanged in an electrically non-conducting carrier fluid. In present study, the considered ferrite nanoparticles are MnZnFe_2_O_4_ (manganese zinc ferrite), Fe_2_O_4_ (magnetite ferrite), and NiZnFe_2_O_4_ (nickel zinc ferrite) [11, 12] crystallizes in the normal spinal structure. The carrier fluid is taken to be water (H_2_O) and ethylene glycol (C_2_H_6_O_2_). In ferromagnetic nanofluids hyperthermia, ferrites nanoparticles of various types like MnZnFe_2_O_4_, Fe_2_O_4_, and NiZnFe_2_O_4_ or even of hematite are infused in tumor and afterward subjected under a high frequency magnetic field. These ferrite nanoparticles produce heat that regularly enhances tumor temperature, which can kill cancer cell [13] A well-tempered of these ferrites are, therefore, characterized by containing the iron atoms situated at the origins of octahedra of oxygen atoms and zinc atoms originated in tetrahedra of oxygen atoms. Characteristically, the normal spinels are paramagnetic and the inverted spinels are ferromagnetic at the room temperature. Further, at low temperature zinc ferrites are behave like antiferromagnetic. Ferrofluids do not hold magnetization in the absence of a magnetic dipole and are classified into superparamagnets. A remarkable feature of the ferromagnetic nanofluids is the reliance of magnetization on the temperature and this thermomagnetization coupling makes ferromagnetic nanofluids more applicable in various practical applications [14–17]. Ferrofluids can be used to capture magnetic domain structures on the surface of ferrofluids in presence of magnetic dipole utilizing a procedure introduced by Mee [18]. The flow of a ferrofluid under the impact of magnetic field and thermal gradients are explored by Neuringer [19]. Nadeem *et* *al*. [20] depicted the influence of a magnetic dipole with porous medium in the flow of a ferrofluid. Anderson and Valnes [21] analyzed the effects of a magnetic field produced by magnetic dipole over a stretched sheet (shear driven motion) and concluded that the magnetic field is responsible for slow downing the motion of fluid. Zeeshan and Majeed demonstrated the influence of the magnetic dipole and suction/injection in a Jeffrey fluid flow over a stretchable surface [22]. Heat transfer analysis in a ferromagnetic fluid flow over a stretching surface is exposed by Majeed *et* *al*. [23]. Some applications relevant to the flow of liquids may be found in [24–35].

The purpose of the article is to exhibit theoretically the practicability of the concept of ferromagnetic nanoparticles with Fe_2_O_4_ (magnetite ferrite), NiZnFe_2_O_4_ (Nickel zinc ferrite), and MnZnFe_2_O_4_ (manganese zinc ferrite) as ferrites nanoparticles and C_2_H_6_O_2_ (ethylene glycol) and H_2_O (water) as base fluid. The present analysis concentrates on depicting the heat transport phenomenon in the flow of ferromagnetic nanofluids. A comparison has been made for different ferrites nanoparticles in the analysis of axial velocity, temperature field, wall shear stress and heat transfer rate. The constitutive equations for velocity and temperature are taken under the boundary layer assumptions. In the wake of utilizing appropriate similarity variables, the final form of boundary value problem is clarified numerically with the help of BVPh2—midpoint method and analytically with optimal homotopy analysis method. The physical emerging parameters are portrayed through tables and graphs.

## 2 Ferrohydrodynamic and thermal energy equations

### 2.1 Flow analysis

**Fig 1:**
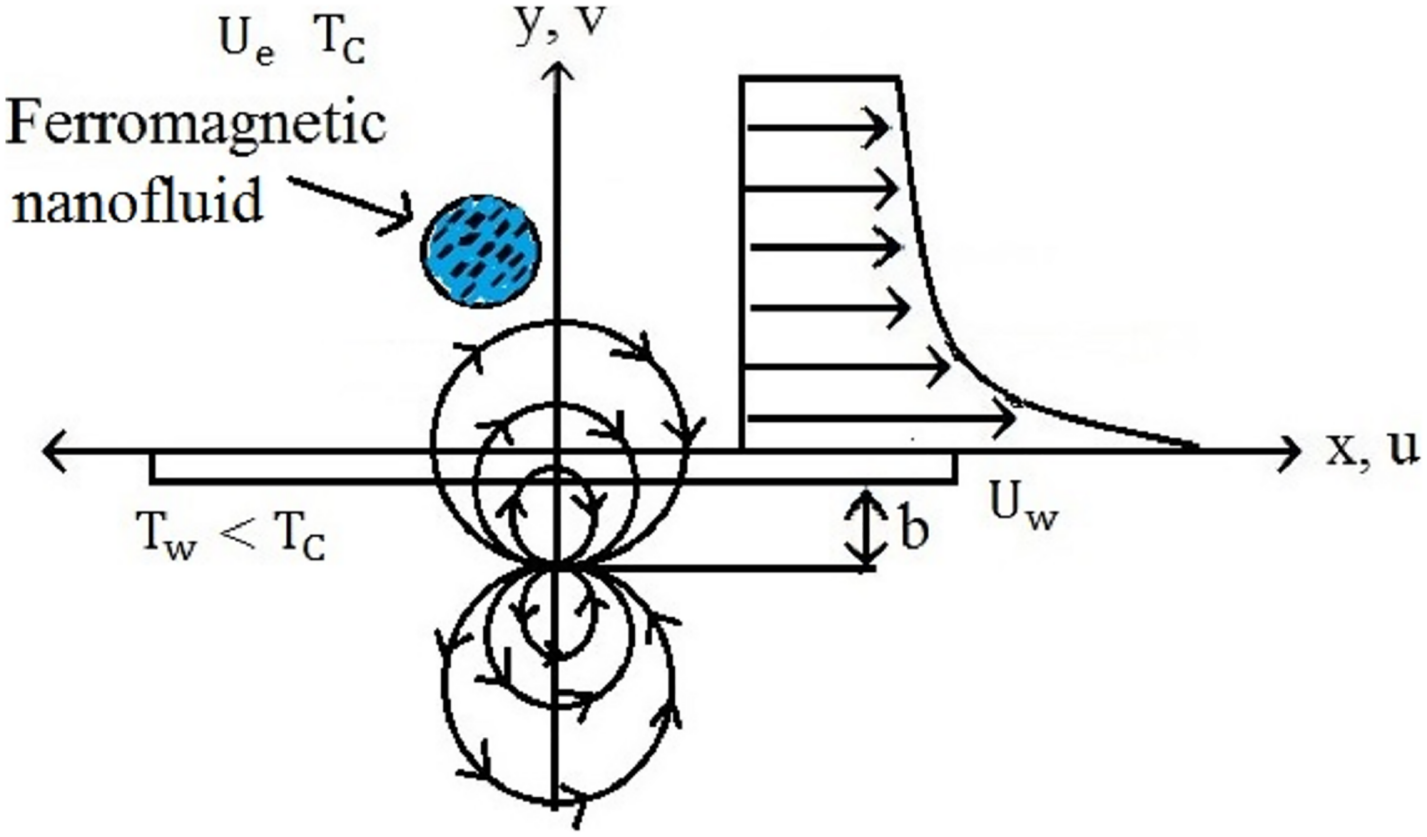
Geometry of the flow problem.

Consider an electrically non—conducting, steady, an incompressible and laminar viscous boundary layer flow of a ferromagnetic MnZnFe_2_O_4_—C_2_H_6_O_2_ (manganese zinc ferrite-ethylene glycol), NiZnFe_2_O_4_—C_2_H_6_O_2_ (Nickel zinc ferrite-ethylene glycol), Fe_2_O_4_—C_2_H_6_O_2_ (magnetite ferrite-ethylene glycol), NiZnFe_2_O_4_—H_2_O (Nickel zinc ferrite-water), MnZnFe_2_O_4_—H_2_O (manganese zinc ferrite-water), and Fe_2_O_4_—H_2_O (magnetite ferrite-water) nanofluids along a continuously stretching surface. The effect of the magnetic dipole is taken in such a way that its center exactly lies on the *x*—axis. The nanofluid flow is caused due to the stretching of the sheet. The velocity of the stretching sheet is *U*_*w*_ = *Sx* (*S* is a dimensionless constant) and *T* = *T*_*w*_ and *T* = *T*_∞_ symbolizes the respective temperature at the stretching sheet and ambient fluid. The magnetic field points of magnetic dipole are applied in positive *x*—direction. To make ferrofluid saturate, the magnetic dipole improve the magnetic field of significant strength. The geometry for the flow evaluation is shown in Fig 1. The fluid above Curie temperature *T*_*c*_ is not capable of being magnetized. It is assumed that the Curie temperature is greater than the temperature at stretching sheet, instead, the temperature *T* = *T*_∞_ is supposed to be temperature of the fluid away from the surface, where *T*_*w*_ < *T*_∞_ < *T*_*c*_. It is presumed that the nanoparticles and base fluids are in thermal equilibrium and occurs no slip between them. The thermophysical properties of the nanofluids MnZnFe_2_O_4_—C_2_H_6_O_2_, NiZnFe_2_O_4_—C_2_H_6_O_2_, and Fe_2_O_4_—C_2_H_6_O_2_ are taken as in Table 1. Considering the above assumptions into account, applying the boundary layer approximation *O* (*u*) = *O* (*x*) = *O* (1) and *O* (*v*) = *O* (*y*) = *O* (*δ*), the boundary layer equations in a ferrohydrodynamic and thermal energy equations are

$$\begin{array}{c} \frac{\partial u}{\partial x}+\frac{\partial v}{\partial y}=0, \end{array}$$ (1)

$$\begin{array}{c} \rho_{nf}(u\frac{\partial u}{\partial x}+v\frac{\partial u}{\partial y})=-\frac{\partial p}{\partial x}+\mu_{0}M\frac{\partial H}{\partial x}+\mu_{nf}\frac{\partial^{2}u}{\partial y^{2}}-\frac{\upsilon_{nf}\epsilon }{K_{1}}u, \end{array}$$ (2)

$$\begin{array}{c} {\left(\rho c_{p}\right)}_{nf}\left(u\frac{\partial T}{\partial x}+v\frac{\partial T}{\partial y}\right)+\left(u\frac{\partial H}{\partial x}+v\frac{\partial H}{\partial y}\right)\mu_{0}T\frac{\partial M}{\partial T}=k_{nf}\frac{\partial^{2}T}{\partial y^{2}}, \end{array}$$ (3)

where (*u*, *v*) identify the respective components of velocity along (*x*, *y*) directions, *μ*_0_ signify the magnetic permeability, *P* designate pressure, *μ*_*nf*_ exemplify the dynamic viscosity of nanofluid, *ρ*_*nf*_ indicate nanofluid density, *υ*_*nf*_ specify the kinematic viscosity of nanofluid, *K*_1_ and *ϵ* are the respective permeability and porosity of porous medium, (*ρc*_*p*_)_*nf*_ display the specific heat, *T* delegate the temperature, *k*_*nf*_ identify thermal conductivity of the nanofluid, *H* communicate the magnetic field, and *M* exemplify the magnetization.

**Table 1:** Thermo-physical properties of ethylene glycol, water, manganese zinc ferrite, nickel zinc ferrite, and magnetite ferrite.

	*ρ*(kg/m^3^)	*C*_*p*_(J/kgK)	*k*(W/mK)	Pr
Ethylene glycol(C_2_H_6_O_2_)	1116.6	2382	0.249	204
Water(H_2_O)	998.3	4182	0.60	6.96
Nickel zinc ferrite(NiZnFe_2_O_4_)	4800	710	6.3	—
Manganese zinc ferrite(MnZnFe_2_O_4_)	4700	1050	3.9	—
Magnetite ferrite(Fe_2_O_4_)	5180	670	9.7	—

The admissible boundary conditions for the boundary value problem are assumed to be of the form

$$\begin{array}{l} {u|}_{y=0}=U_{w}=Sx,\;{v|}_{y=0}=0,\;{\frac{\partial T}{\partial y}|}_{y=0}=-h_{c}T,\;,\\ {u|}_{y\to \infty }\to 0,\;{T|}_{y\to \infty }\to T_{\infty }=T_{c}. \end{array}$$ (4)

In above Eq 4, *U*_*w*_ exemplify the stretching velocity, the temperature condition recommended at *y* = 0 speaks to the Newtonian heating effects, *h*_*c*_ signify heat transfer coefficient, and *y* → ∞ describes the Curie temperature *T*_*c*_ at the boundaries, *T*_∞_ signify temperature of ambient fluid.

### 2.2 Thermo-physical properties of MnZnFe_2_O_4_-C_2_H_6_O_2_, NiZnFe_2_O_4_-C_2_H_6_O_2_, Fe_2_O_4_-C_2_H_6_O_2_, MnZnFe_2_O_4_-H_2_O, NiZnFe_2_O_4_-H_2_, and Fe_2_O_4_-H_2_O nanofluids

The dynamic viscosity *μ*_*nf*_, the effective dynamic density *ρ*_*nf*_, the specific heat or heat capacitance (*ρc*_*p*_)_*nf*_, and the thermal conductivity *k*_*nf*_ of the nanofluid are given by

$$\begin{array}{ccc} & & \rho_{nf}=\left(1-\varphi \right)\rho_{f}+\varphi \rho_{s},\;\frac{\mu_{nf}}{\mu_{f}}=\frac{1}{{\left(1-\varphi \right)}^{2.5}},\\ & & {\left(\rho C_{p}\right)}_{nf}=\left(1-\varphi \right){\left(\rho C_{p}\right)}_{f}+\varphi {\left(\rho C_{p}\right)}_{s},\\ & & \frac{k_{nf}}{k_{f}}=\frac{\left(k_{s}+2k_{f}\right)-2\varphi \left(k_{f}-k_{s}\right)}{\left(k_{s}+2k_{f}\right)+\varphi \left(k_{f}-k_{s}\right)}. \end{array}$$ (5)

Eq 5 is the general relationship used to calculate the density *ρ*_*nf*_, dynamic viscosity *μ*_*nf*_, specific heat (*ρC*_*p*_)_*nf*_, and thermal conductivity *k*_*nf*_ for nanofluids. Where *k*_*s*_ and *k*_*f*_ are the respective thermal conductivities of the base fluid and nanoparticle, and *φ* is the solid volume fraction of nanofluid, *ρ*_*s*_ and *ρ*_*f*_ are the respective densities of the nano-particle and base fluid. The thermo-physical properties of of the present analysis are listed in Table 1.

### 2.3 Magnetic dipole

The flow of ferrofluid induced by stretching sheet is influenced by the magnetic field due to the magnetic dipole. Magnetic scalar potential δ* portray the region of a magnetic dipole, which is defined as

$$\begin{array}{c} \delta^{*}=\frac{\gamma_{1}}{2\pi }\frac{x}{x^{2}+{\left(y+b\right)}^{2}}, \end{array}$$ (6)

here *γ*_1_ symbolize the strength of magnetic field at the source and *b* is the distance from the center of magnetic field to *x*-axis. The components for the magnetic field (*H*) are

$$\begin{array}{c} H_{x}=-\frac{\partial \delta^{*}}{\partial x}=\frac{\gamma_{1}}{2\pi }\frac{x^{2}-{\left(y+b\right)}^{2}}{{\left(x^{2}+{\left(y+b\right)}^{2}\right)}^{2}}, \end{array}$$ (7)

$$\begin{array}{c} H_{y}=-\frac{\partial \delta^{*}}{\partial y}=\frac{\gamma_{1}}{2\pi }\frac{2x\left(y+c\right)}{{\left(x^{2}+{\left(y+c\right)}^{2}\right)}^{2}}. \end{array}$$ (8)

Since the magnetic body force is proportional to the gradient of the magnitude of *H*, we obtain

$$\begin{array}{c} H=\sqrt{{\left(\frac{\partial \delta^{*}}{\partial x}\right)}^{2}+{\left(\frac{\partial \delta^{*}}{\partial y}\right)}^{2}}. \end{array}$$ (9)

Making use of Eqs 6 and 7 in Eq 8, we get the resulting equations, after reached out in powers of *x* and held terms up to organize *x*^2^,

$$\begin{array}{c} \frac{\partial H}{\partial x}=-\frac{\gamma_{1}}{2\pi }\frac{2x}{{\left(y+b\right)}^{4}}, \end{array}$$ (10)

$$\begin{array}{c} \frac{\partial H}{\partial y}=\frac{\gamma_{1}}{2\pi }(-\frac{2}{{\left(y+b\right)}^{3}}+\frac{4x^{2}}{{\left(y+b\right)}^{5}}). \end{array}$$ (11)

The influence of magnetization *M* with temperature *T* is defined by the linear expression below,

$$\begin{array}{c} M=K_{2}\left(T-T_{\infty }\right), \end{array}$$ (12)

here *K*_2_ identifies the pyromagnetic coefficient. The geometry of a heated ferrofluid appears in Fig 1. Here the round lines exhibits the magnetic field.

## 3 Solution procedure

Here we introduce the nondimensional variables as exposed by Andersson [9]

$$\begin{array}{l} \psi \left(\eta ,\xi \right)=\left(\frac{\mu_{f}}{\rho_{f}}\right)\eta f\left(\xi \right),\\ \theta \left(\eta ,\xi \right)\equiv \frac{T_{c}-T}{T_{c}-T_{w}}=\theta_{1}\left(\xi \right)+\eta^{2}\theta_{2}\left(\xi \right), \end{array}$$ (13)

in which *μ*_*f*_ represents the dynamic viscosity, *θ*_1_(*η*, *ξ*) and *θ*_2_(*η*, *ξ*) displays the dimensionless temperature, the corresponding non—dimensional coordinates are

$$\begin{array}{c} \xi =y{\left(\frac{\rho_{f}S}{\mu_{f}}\right)}^{1/2},\;\;\;\eta =x{\left(\frac{\rho_{f}S}{\mu_{f}}\right)}^{1/2}. \end{array}$$ (14)

The stream function are delineated in such a way that the continuity equation is directly satisfied, the comparable velocity components *u* and *v* are defined as follow

$$\begin{array}{c} u=\frac{\partial \psi }{\partial y}=Sxf^{\prime }\left(\xi \right),\;\;\;v=-\frac{\partial \psi }{\partial x}=-{\left(S\upsilon_{f}\right)}^{1/2}f\left(\xi \right), \end{array}$$ (15)

here prime denotes differentiation with respect to *ξ*. Making use of the dimensionless variables defined in Eqs (16–18), the Eqs (2) and (3) along with admissible boundary conditions are given in Eq (4) reduces to the following form of coupled equations and corresponding boundary conditions

$$\begin{array}{c} \frac{1}{{\left(1-\varphi \right)}^{2.5}\left(1-\varphi +\varphi \frac{\rho_{s}}{\rho_{f}}\right)}f^{\prime \prime \prime }-f^{\prime 2}+ff^{\prime \prime }-\frac{2\beta \theta_{1}}{\left(1-\varphi +\varphi \frac{\rho_{s}}{\rho_{f}}\right){\left(\xi +\gamma \right)}^{4}}-\frac{P_{m}}{\left(1-\varphi +\varphi \frac{\rho_{s}}{\rho_{f}}\right)}f^{\prime }=0, \end{array}$$ (16)

$$\begin{array}{c} \frac{k_{nf}/k_{f}}{\left(1-\varphi +\varphi \frac{{\left(\rho C_{p}\right)}_{s}}{{\left(\rho C_{p}\right)}_{f}}\right)}\theta_{1}^{\prime \prime }+\text{Pr}\left(f\theta_{1}^{\prime }-2f^{\prime }\theta_{1}\right)+\frac{2\lambda \beta f\left(\theta_{1}-\varepsilon \right)}{{\left(\xi +\gamma \right)}^{3}}-4\lambda f^{\prime 2}=0, \end{array}$$ (17)

$$\begin{array}{l} \frac{k_{nf}/k_{f}}{\left(1-\varphi +\varphi \frac{{\left(\rho C_{p}\right)}_{s}}{{\left(\rho C_{p}\right)}_{f}}\right)}\theta_{2}^{\prime \prime }-\text{Pr}\left(4f^{\prime }\theta_{2}-f\theta_{2}^{\prime }\right)+\frac{2\lambda \beta f\theta_{2}}{{\left(\xi +\gamma \right)}^{3}}\\ -\lambda \beta \left(\theta_{1}-\varepsilon \right)\left(\frac{2f^{\prime }}{{\left(\xi +\gamma \right)}^{4}}+\frac{4f}{{\left(\xi +\gamma \right)}^{5}}\right)-\lambda f^{\prime \prime 2}=0, \end{array}$$ (18)

$$\begin{array}{c} f\left(\xi \right)=0,\;f^{\prime }\left(\xi \right)=1,\;\theta_{1}^{\prime }\left(\xi \right)=-\lambda_{1}\left(1+\theta_{1}\left(0\right)\right),\;\theta_{2}\left(\xi \right)=0,\;\;\;\text{at}\;\xi =0, \end{array}$$ (19)

$$\begin{array}{c} f^{\prime }\left(\xi \right)\to 0,\;\theta_{1}\left(\xi \right)\to 0,\;\theta_{2}\left(\xi \right)\to 0,\;\;\;\text{when}\;\xi \to \infty . \end{array}$$ (20)

In above system of nonlinear equations, the parameters λ (viscous dissipation), λ_1_ (the conjugate parameter of Newtonian heating), *β* (ferrohydrodynamic interaction), *P*_*m*_ (porosity parameter), *ε* (Curie temperature) and Pr (Prandtl number) are defined as

$$\begin{array}{ccc} & & \varepsilon =\frac{T_{\infty }}{T_{c}-T_{w}},\lambda_{1}=h_{c}\sqrt{\frac{\upsilon_{f}}{S^{2}}},\;\lambda =\frac{S\mu_{f}^{2}}{\rho K_{2}(T_{c}-T_{w})},\;P_{m}=\frac{\upsilon_{f}\epsilon }{K_{1}S},\\ & & \text{Pr}=\frac{\upsilon_{f}}{\alpha_{f}},\;\beta =\frac{\gamma_{1}}{2\pi }\frac{\mu_{0}K_{2}(T_{c}-T_{w})\rho_{f}}{\mu_{f}^{2}},\;\gamma =\sqrt{\frac{S\rho_{f}b^{2}}{\mu_{f}}}. \end{array}$$ (21)

Skin friction coefficient and local Nusselt number are expressed as

$$\begin{array}{lll} C_{f} & = & \frac{-2\tau_{w}}{\rho_{nf}U_{w}^{2}},\;\tau_{w}=\mu_{nf}\;{\frac{\partial u}{\partial y}|}_{y=0},\\ Nu & = & \frac{xk_{nf}}{k_{f}\left(T_{c}-T_{w}\right)}{\frac{\partial T}{\partial y}|}_{y=0}. \end{array}$$ (22)

The dimensionless equations for the skin friction coefficient and Nusselt number (the ratio of convective to conductive heat transfer coefficients) i.e. local surface heat flux

$$\begin{array}{ccc} \frac{1}{2}{\text{Re}}_{x}^{1/2}C_{f} & = & \frac{1}{{\left(1-\varphi \right)}^{2.5}}f^{\prime \prime }\left(0\right),\\ {\text{Re}}_{x}^{-1/2}Nu_{x} & = & -\frac{\lambda_{1}k_{nf}}{k_{f}}(1+\frac{1}{\theta_{1}\left(0\right)+\zeta^{2}\theta_{2}\left(0\right)}). \end{array}$$ (23)

${\text{Re}}_{x}^{1/2}C_{f}$ is the local skin friction coefficient and ${\text{Re}}_{x}^{-1/2}Nu_{x}$ is the Nusselt number, in which Re_*x*_ = *xU*_*w*_(*x*)/*υ*_*f*_ = *Sx*^2^/*υ*_*f*_ is a local Reynolds number (i.e. the ratio of inertial to viscous forces) depends on the stretching velocity *U*_*w*_(*x*).

BVPh2—Midpoint method (Maple) and optimal homotopy analysis method (Mathematica 9.0) are implemented in the present analysis for the solution of the non-linear ordinary momentum Eq 16 and thermal energy Eqs 17 and 18 subjected to the admissible boundary conditions in Eqs 19 and 20. These techniques are utilized to get the solutions for highly non—linear equations. The optimal HAM [36, 37] gives better results as compared to perturbation techniques and other conventional investigative techniques. The generality of the optimal HAM often allows for good convergence of the solution over larger spatial and parameter domains. Firstly, the optimal HAM gives us a remarkable flexibility to pick the equation type of linear sub-problems. Secondly, the optimal HAM works regardless of the possibility that there don’t exist any small/large physical parameters in determining equations and boundary/initial conditions. Particularly, unlike perturbation and other analytic techniques, the optimal HAM gives us an advantageous approach to guarantee the convergence of series solution by method of presenting the supposed convergence control parameter into the series solution. Moreover, the optimal HAM utilize the homotopy/auxiliary parameter only on a theoretical level to depict that a nonlinear system of differential equations may be divided into a set of linear system of differential equations which are solved analytically, while the continuation methods require solving a discrete linear system as the homotopy parameter is varied to solve the nonlinear system. The respective linear operators and their relating initial guesses for the boundary value problem are

$$\begin{array}{c} L_{f}\left(f\right)=\frac{d^{3}f}{d\xi^{3}}+\frac{d^{2}f}{d\xi^{2}},L_{\theta_{1}}\left(\theta_{1}\right)=\frac{d^{2}\theta_{1}}{d\xi^{2}}-\theta_{1},L_{\theta_{2}}\left(\theta_{2}\right)=\frac{d^{2}\theta_{2}}{d\xi^{2}}-\theta_{2}, \end{array}$$ (24)

$$\begin{array}{c} f_{0}\left(\xi \right)=1-\text{exp}\left(-\xi \right),\;\theta_{1_{0}}\left(\xi \right)=\frac{\lambda_{1}}{1-\lambda_{1}}\text{exp}\left(-\xi \right), \end{array}$$ (25)

$$\begin{array}{c} \theta_{2_{0}}(\xi )=\xi \text{exp}(-\xi ), \end{array}$$ (26)

where *L*_*f*_ (*f*), *L*_*θ*_1__ (*θ*_1_), and *L*_*θ*_2__ (*θ*_2_) symbolizes the linear operators, on the other hand *f*_0_ (*ξ*), *θ*_1_0__ (*ξ*), and *θ*_2_0__ (*ξ*) illustrate the respective initial guesses of *f*, *θ*_1_, and *θ*_2_.

## 4 Convergence analysis for optimal HAM solution

The auxiliary parameters ℏ_*f*_, ℏ_*θ*_1__, and ℏ_*θ*_2__ have a leading purpose of controlling the convergence of homotopic solutions. To get convergent solutions, we take suggested values of these parameters. For this reason, residual errors are noticed for momentum, and thermal energy equations by initiating the expressions given below,

$$\begin{array}{c} \Delta_{m}^{f}=\int_{0}^{1}{\left[R_{m}^{f}(\xi ,\hbar_{f})\right]}^{2}d\xi , \end{array}$$ (27)

$$\begin{array}{c} \Delta_{m}^{\theta_{1}}=\int_{0}^{1}{\left[R_{m}^{\theta_{1}}(\xi ,\hbar_{\theta_{1}})\right]}^{2}d\xi , \end{array}$$ (28)

$$\begin{array}{c} \Delta_{m}^{\theta_{2}}=\int_{0}^{1}{\left[R_{m}^{\theta_{2}}(\xi ,\hbar_{\theta_{2}})\right]}^{2}d\xi , \end{array}$$ (29)

The convergence of the parametric values is displayed by OHAM, listed in the following Tables 2 and 3, using the values of the parameters *β* = 1.2, λ = 0.01, λ_1_ = 0.5, Pr = 204, *φ* = 0.1, and *γ* = 0.1.

**Table 2:** Shows the average residual square errors ($\Delta_{m}^{t}$).

$\frac{values\to }{order↓}$	ℏ_*f*_	ℏ_*θ*_1__	ℏ_*θ*_2__	$\Delta_{m}^{t}$
4	−0.40025	−0.42903	−0.97231	0.009324
6	−0.44380	−0.49902	−0.98331	2.5489 × 10^−4^
8	−0.54992	−0.49930	−0.99005	1.09211 × 10^−10^
10	−0.60321	−0.54173	−0.92152	3.62310 × 10^−15^
12	−0.76119	−0.76497	−1.10921	7.16290 × 10^−21^

**Table 3:** Shows individual residual square errors for $\Delta_{m}^{f},\Delta_{m}^{\theta_{1}},$ and $\Delta_{m}^{\theta_{2}}$.

$\frac{values\to }{order↓}$	ℏ_*f*_ = −0.76119	ℏ_*θ*_1__ = −0.76497	ℏ_*θ*_2__ = −1.10921
	$\Delta_{m}^{f}$	$\Delta_{m}^{\theta_{1}}$	$\Delta_{m}^{\theta_{2}}$
8	2.54891 × 10^−20^	3.44370 × 10^−10^	9.54570 × 10^−10^
10	6.43670 × 10^−22^	1.45219 × 10^−16^	0.64881 × 10^−14^
12	0.32671 × 10^−25^	8.33670 × 10^−20^	6.45672 × 10^−18^
20	2.34589 × 10^−26^	1.54891 × 10^−25^	2.43370 × 10^−23^

**Fig 2:**
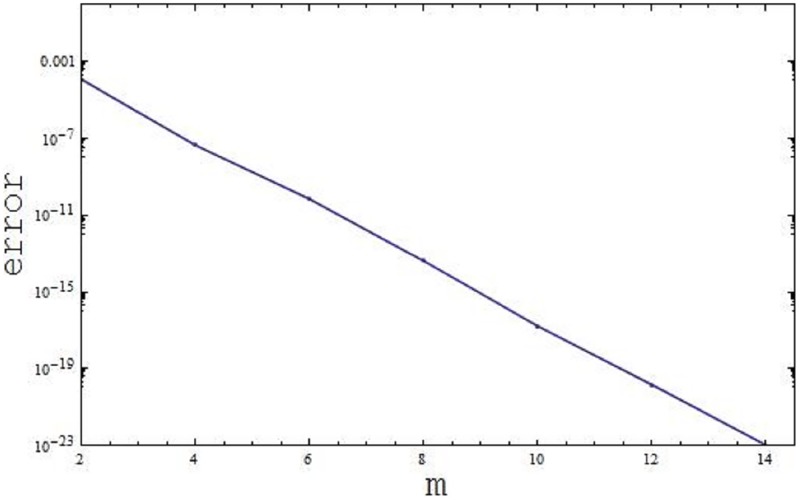
Display the error decay for the 10^*th*^ order approximation.

The graphical representation for the 10^*th*^ order approximation display the error decay in the following Fig 2.

Here $\Delta_{m}^{t}$ indicate the total discrete squared residual error.

$$\begin{array}{c} \Delta_{m}^{t}=\Delta_{m}^{f}+\Delta_{m}^{\theta_{1}}+\Delta_{m}^{\theta_{2}}. \end{array}$$ (30)

Here the $\Delta_{m}^{t}$ is used to obtain the optimal convergence control parameters.

## 5 Results and discussion

This section contains the physical interpretation of sundry parameters on the flow field. The effects of dimensionless emerging parameters *β* (ferrohydrodynamic interaction), *P*_*m*_ (porosity parameter), *φ* (solid volume fraction of nanofluid), λ_1_ (the conjugate parameter of Newtonian heating), and Pr (Prandtl number) are analyzed. Moreover, rest of the materialize parameters in the flow problem are considered fixed. The fixed values of these parameters are taken as λ = 0.01, *ε* = 2.0, *γ*_1_ = 1.0. The boundary value problem is solved numerically and analytically via BVPh2-Midpoint method and optimal homotopy analysis method (OHAM) respectively. The accuracy of the present optimal HAM and BVPh2—midpoint method is tested by comparing $\theta_{1}^{\prime }(0)$ values with those of Rashidi [38] for pure fluid that is tabulated in Table 4. An excellent agreement between the result is found for special case of the present problem. The boundary layer flow of a ferromagnetic NiZnFe_2_O_4_—C_2_H_6_O_2_, NiZnFe_2_O_4_—H_2_O, MnZnFe_2_O_4_—C_2_H_6_O_2_, MnZnFe_2_O_4_—H_2_O, Fe_2_O_4_—C_2_H_6_O_2_, and Fe_2_O_4_—H_2_O nanofluids with nanoparticles are investigated. In order to get an obvious insight of the existing flow problem, the results are dig out for the axial velocity, temperature field, skin friction coefficient, and Nusselt number graphically. The analysis is carried out in the presence of magnetic dipole.

**Table 4:** Comparison of Nusselt number for the case when *β* = λ = *ε* = *γ* = 0.

Pr	Rashidi [38]	$\frac{\text{OHAM results}}{{\text{Re}}_{x}^{-1/2}X^{-1}Nu_{x}}$	$\frac{\text{BVPh2-Midpoint}}{{\text{Re}}_{x}^{-1/2}X^{-1}Nu_{x}}$
0.72	0.808631	0.808641	0.808639
1.0	1.000000	1.000000	1.000000
3.0	1.923682	1.923690	1.923672
4.0	− − −	2.003170	2.003162
5.0	− − −	2.329810	2.329871
8.0	− − −	− − −	2.541990

**Fig 3:**
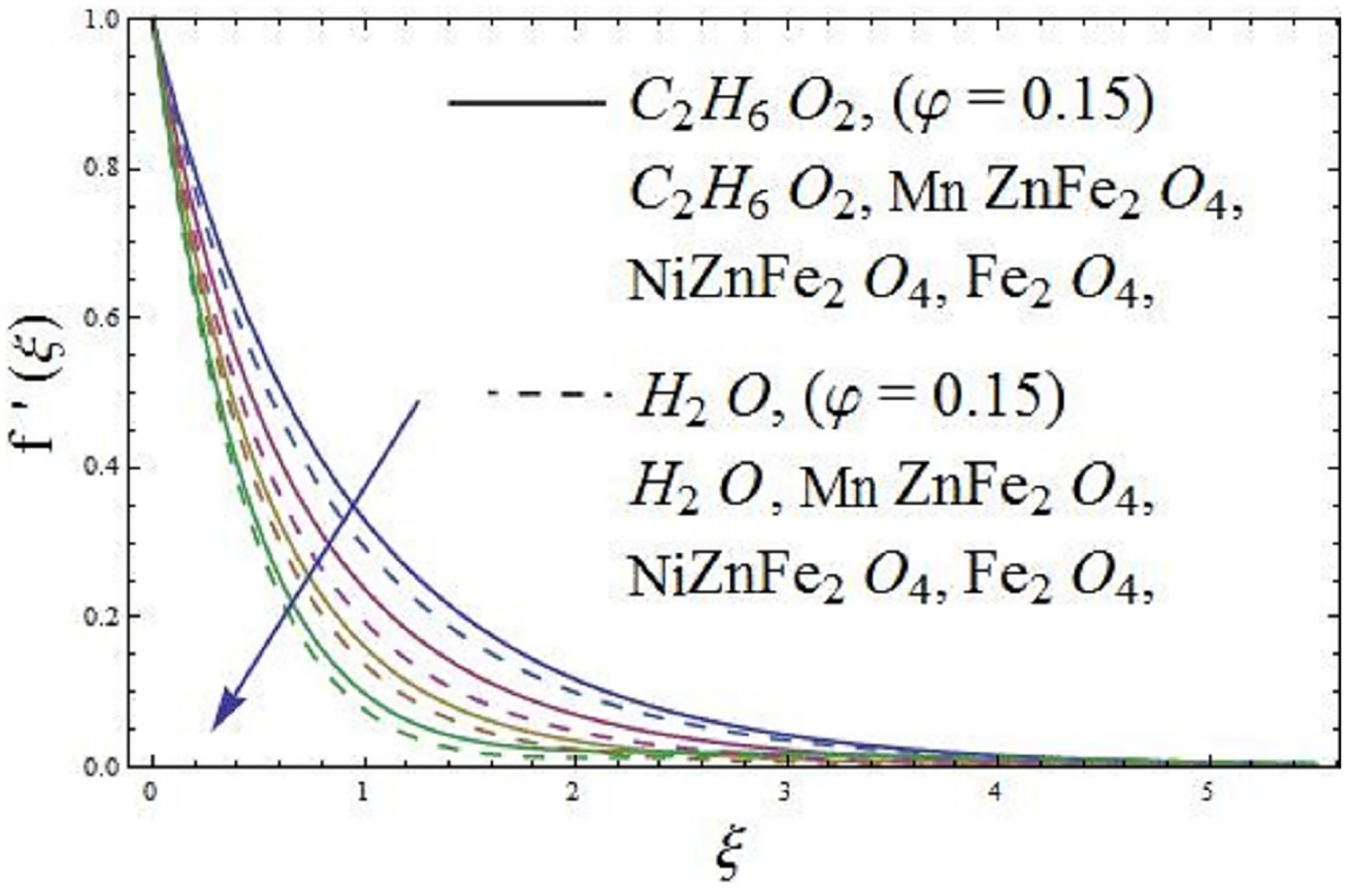
The effect of parameter *φ* (solid volume fraction) on axial velocity.

**Fig 4:**
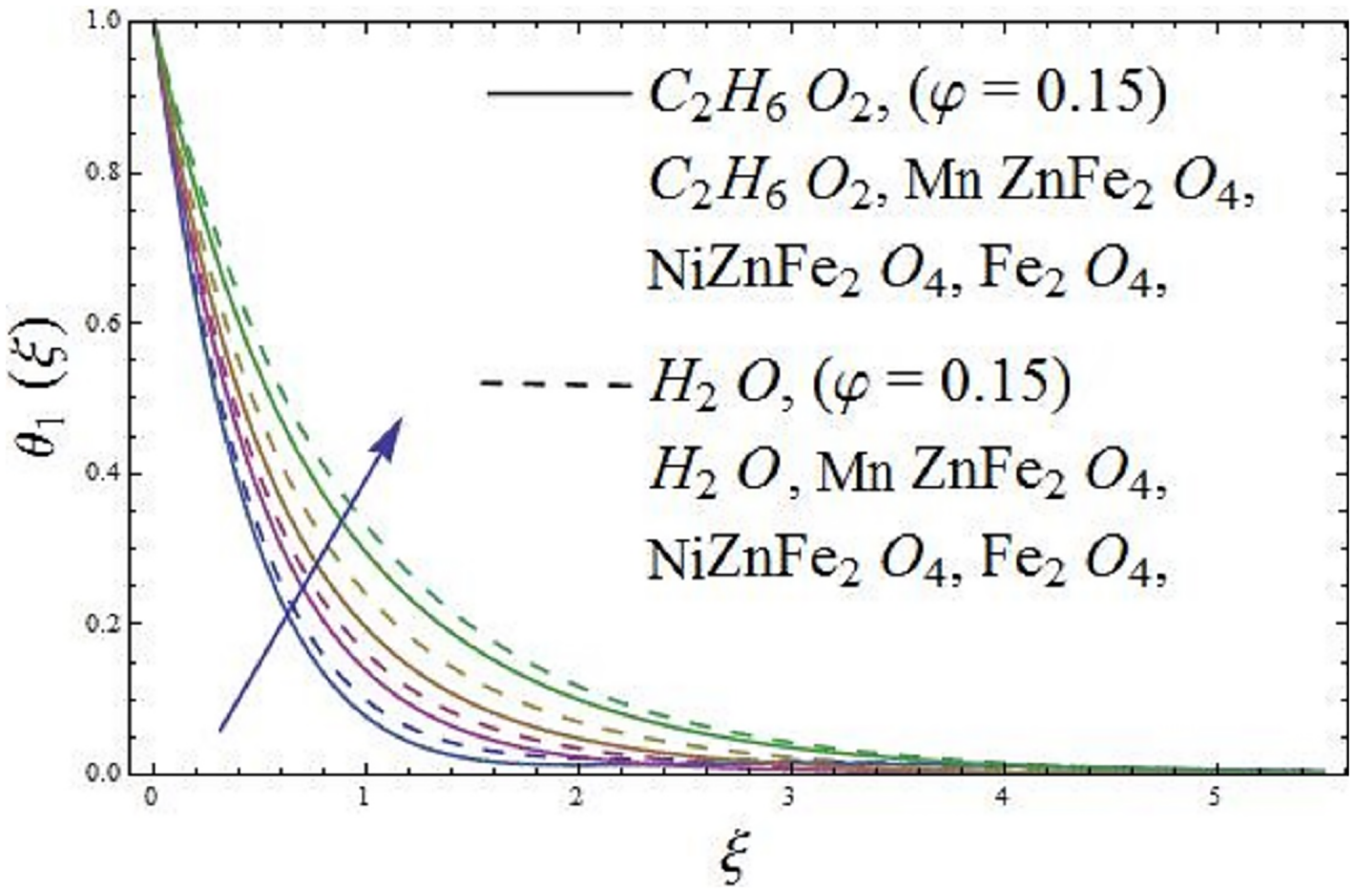
The effect of parameter *φ* (solid volume fraction) on temperature field.

The influence of parameter *φ* (solid volume fraction of nanofluid) of the ferromagnetic NiZnFe_2_O_4_—C_2_H_6_O_2_, NiZnFe_2_O_4_—H_2_O, MnZnFe_2_O_4_—C_2_H_6_O_2_, MnZnFe_2_O_4_—H_2_O, Fe_2_O_4_—C_2_H_6_O_2_, and Fe_2_O_4_—H_2_O nanofluids are depicted in Figs 3 and 4 on the dimensionless axial velocity and temperature field. It is evident from Fig 3 that the axial velocity of the respective nanofluids decreases with an increase in parameter *φ* (solid volume fraction of nanofluid). The axial velocity reduces as we move far away from the surface. In fact, an increase in parameter *φ* (solid volume fraction of nanofluid) causes to concentrates the ferromagnetic fluid which consequently produces resistance to the fluid motion and as a result, the axial velocity reduces for both the base fluids, i.e., (water and ethylene glycol). The presence of magnetic dipole provides attraction to the ferrites nanoparticles due to which the axial velocity of the ferromagnetic nanofluids slows down. It means that magnetic dipole plays a vital role in reducing the movements of fluid particles. Further, Fig 3 depicts that Fe_2_O_4_ (magnetite ferrite) nanoparticles are more magnetized as compared to NiZnFe_2_O_4_ (Nickel zinc ferrite) and MnZnFe_2_O_4_ (Manganese zinc ferrite) nanoparticles. The more magnetization, the more will be resistance produced by the magnetic dipole to the fluid particles, as a result, it is depicted that Fe_2_O_4_—C_2_H_6_O_2_ and Fe_2_O_4_—H_2_O ferromagnetic nanofluids have low velocity as compared to the ferromagnetic NiZnFe_2_O_4_—C_2_H_6_O_2_, NiZnFe_2_O_4_—H_2_O, MnZnFe_2_O_4_—C_2_H_6_O_2_, and MnZnFe_2_O_4_—H_2_O nanofluids. The characteristics of parameter *φ* (solid volume fraction of nanofluid) on temperature field of the ferromagnetic NiZnFe_2_O_4_—C_2_H_6_O_2_, NiZnFe_2_O_4_—H_2_O, MnZnFe_2_O_4_—C_2_H_6_O_2_, MnZnFe_2_O_4_—H_2_O, Fe_2_O_4_—C_2_H_6_O_2_, and Fe_2_O_4_—H_2_O nanofluids in presence of magnetic dipole are delineated in Fig 4. It is illustrated that temperature field of Fe_2_O_4_—C_2_H_6_O_2_ and Fe_2_O_4_—H_2_O is higher than NiZnFe_2_O_4_—C_2_H_6_O_2_, NiZnFe_2_O_4_—H_2_O, MnZnFe_2_O_4_—C_2_H_6_O_2_, and MnZnFe_2_O_4_—H_2_O nanofluids in presence of magnetic dipole. It is due to the fact that the thermal conductivity of Fe_2_O_4_ (magnetite ferrite) nanoparticles is higher than the thermal conductivity of NiZnFe_2_O_4_ (Nickel zinc ferrite) and MnZnFe_2_O_4_ (manganese zinc ferrite) nanoparticles. Moreover, the presence of magnetic dipole makes higher the temperature field until the temperature of the fluid reach to the Curie temperature *T*_*c*_ of the fluid. It is due to the fact that magnetic dipole produces more resistance to the Fe_2_O_4_ (magnetite ferrite) nanoparticles as compared to NiZnFe_2_O_4_ (Nickel zinc ferrite) and MnZnFe_2_O_4_ (manganese zinc ferrite) nanoparticles, which results in the enhancement of temperature field. If the temperature of the ferrite nanoparticles is higher than the Curie temperature *T*_*c*_ then these ferrite nanoparticles lose their magnetization and there will be no attraction for ferrite nanoparticles whose temperature is higher than the Curie temperature *T*_*c*_.

**Fig 5:**
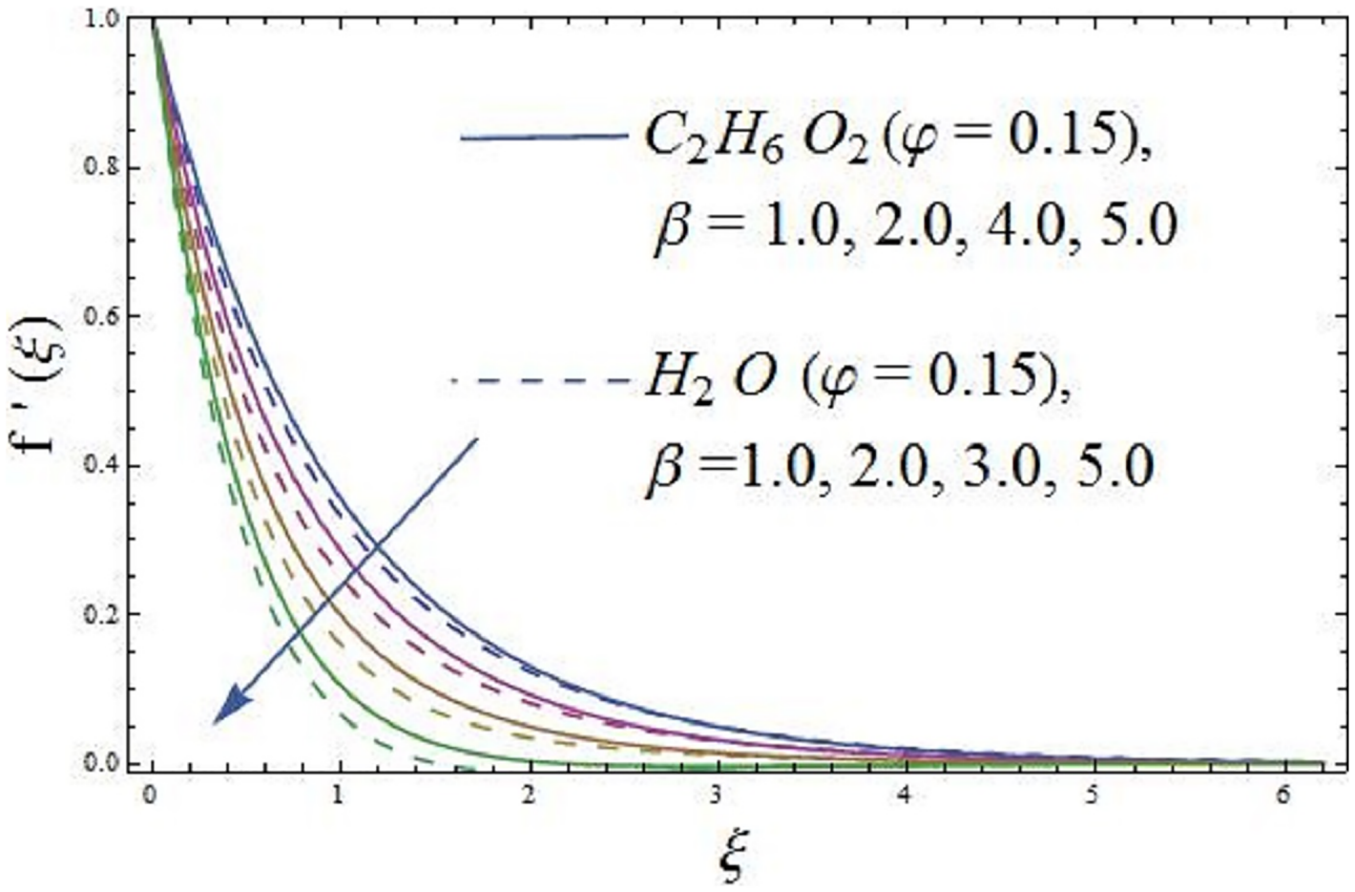
The effect of parameter *β* (ferrohydrodynamic interaction) on axial velocity.

**Fig 6:**
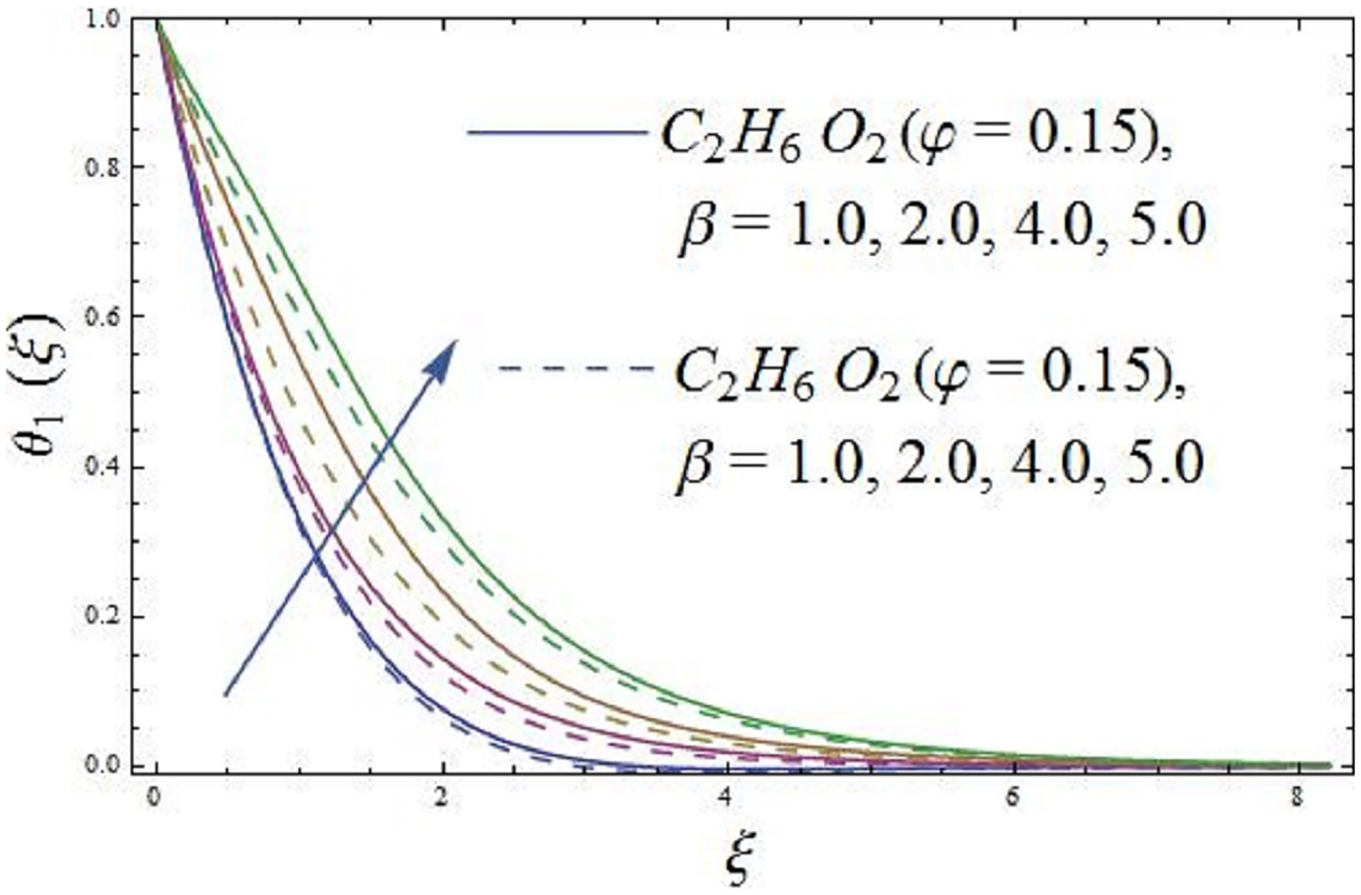
The effect of parameter *β* (ferrohydrodynamic interaction) on temperature field.

The impact of parameter *β* (ferrohydrodynamic interaction) is displayed in Figs 5 and 6. The existence of parameters *γ*_1_ (dimensionless distance from origin to center of magnetic dipole), *ε* (Curie temperature), and *β* (ferrohydrodynamic interaction) is necessary to hold the impact of ferromagnetic effect on the boundary layer flow. The presence of Fe_2_O_4_ (magnetite ferrite), NiZnFe_2_O_4_ (Nickel zinc ferrite) and MnZnFe_2_O_4_ (manganese zinc ferrite) nanoparticles in a viscous carrier fluid corresponds to ferromagnetic nanofluid, because of which viscosity of the fluid enhances and as a result, the axial velocity reduces for enlarging values of parameter *β* (ferrohydrodynamic interaction) as shown in Fig 5. The influence of *β* (ferrohydrodynamic interaction) on axial velocity is carried out for the ferromagnetic NiZnFe_2_O_4_—C_2_H_6_O_2_, NiZnFe_2_O_4_—H_2_O, MnZnFe_2_O_4_—C_2_H_6_O_2_, MnZnFe_2_O_4_—H_2_O, Fe_2_O_4_—C_2_H_6_O_2_, and Fe_2_O_4_—H_2_O nanofluids. It is noticed that the presence of magnetic dipole makes a rapid reduction in the axial velocity of the ferromagnetic nanofluids when water is used as base fluid. The physical interpretation is that the magnetic dipole attracts the ferrite Fe_2_O_4_ (magnetite ferrite), NiZnFe_2_O_4_ (Nickel zinc ferrite) and MnZnFe_2_O_4_ (manganese zinc ferrite) nanoparticles which result in the enhancement of the viscosity of the nanofluid inside the boundary layer and as a result, the axial velocity slows down. The highest velocity is observed for the C_2_H_6_O_2_ (ethylene glycol, when *φ* = 0) and H_2_O (water, when *φ* = 0), whereas the lowest axial velocity is observed for the Fe_2_O_4_—C_2_H_6_O_2_ (magnetite ferrite-ethylene glycol, when *φ* = 0.15) and Fe_2_O_4_—H_2_O (magnetite ferrite-water, when *φ* = 0.15) nanofluids as evident in Fig 5. Fig 6 characterizes the influence of parameter *β* (ferrohydrodynamic interaction) on temperature field. It is depicted that the larger values of parameter *β* (ferrohydrodynamic interaction) leads to enhance the temperature of the nanofluid in presence of the magnetic dipole. It is because of the interaction between an action of a magnetic field and movements of Fe_2_O_4_ (magnetite ferrite), NiZnFe_2_O_4_ (Nickel zinc ferrite) and MnZnFe_2_O_4_ (manganese zinc ferrite) nanoparticles. The interaction between magnetic field action and Fe_2_O_4_ (magnetite ferrite), NiZnFe_2_O_4_ (Nickel zinc ferrite) and MnZnFe_2_O_4_ (manganese zinc ferrite) nanoparticles thinning the axial velocity thereby enhancing frictional heating among fluid layers, that leads to rise thermal boundary layer i.e., the reduction in movements of Fe_2_O_4_ (magnetite ferrite), NiZnFe_2_O_4_ (Nickel zinc ferrite) and MnZnFe_2_O_4_ (manganese zinc ferrite) nanoparticles results in the enhancement of temperature field.

**Fig 7:**
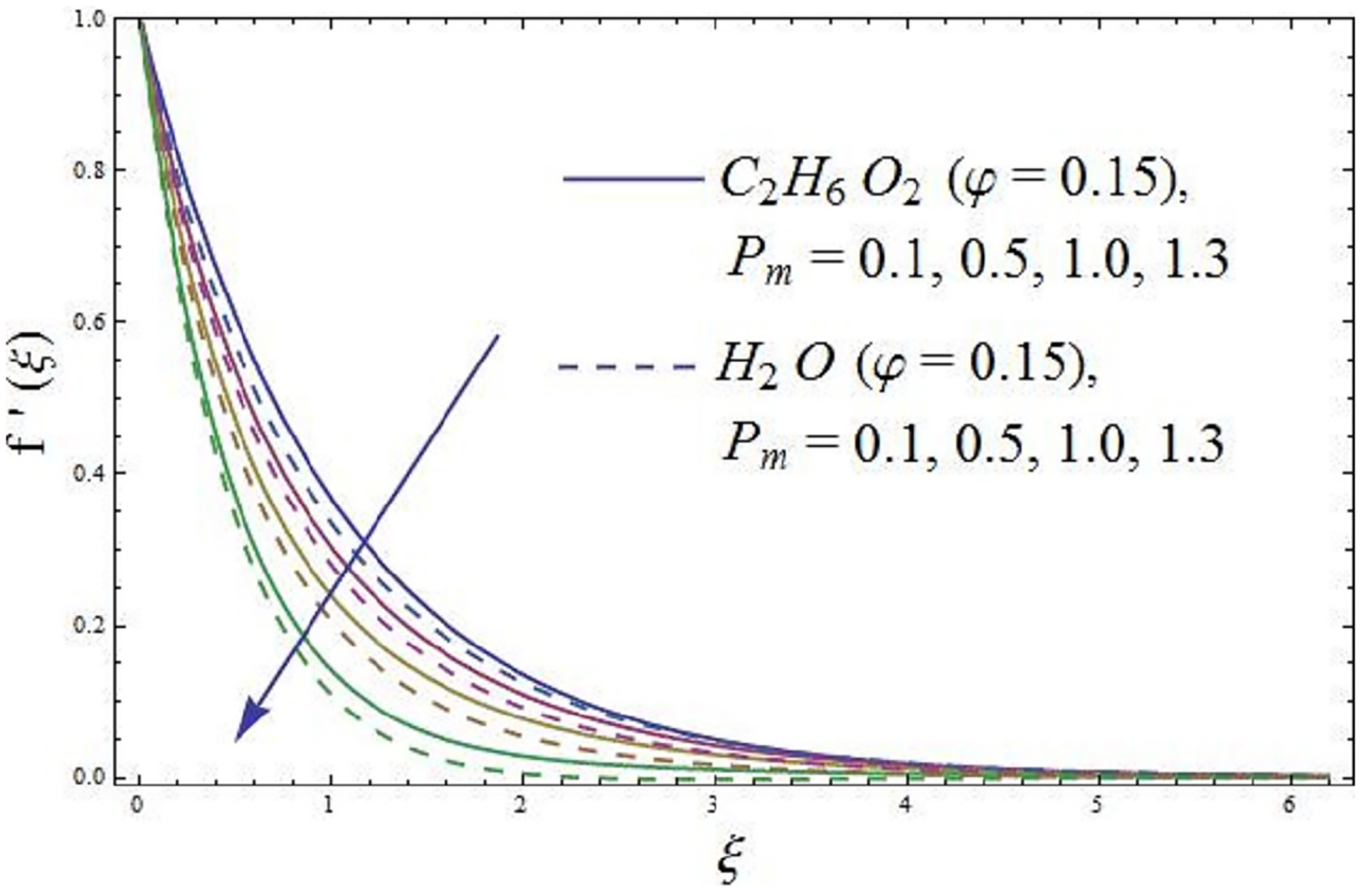
Impact of *P*_*m*_ (Porosity parameter) on axial velocity.

The effect of parameter *P*_*m*_ (Porosity) in the flow of ferromagnetic NiZnFe_2_O_4_—C_2_H_6_O_2_, NiZnFe_2_O_4_—H_2_O, MnZnFe_2_O_4_—C_2_H_6_O_2_, MnZnFe_2_O_4_—H_2_O, Fe_2_O_4_—C_2_H_6_O_2_, and Fe_2_O_4_—H_2_O nanofluids is observed in Fig 7. The existence of parameters *P*_*m*_ (Porosity) in the presence of Fe_2_O_4_ (magnetite ferrite), NiZnFe_2_O_4_ (Nickel zinc ferrite) and MnZnFe_2_O_4_ (manganese zinc ferrite) nanoparticles in a viscous carrier ferromagnetic nanofluid slow down the axial velocity and as a result the axial velocity reduces for enlarging values of parameter *P*_*m*_ (Porosity) as shown in Fig 7. It is depicted that for ferrites-water based ferromagnetic nanofluid in the presence of magnetic dipole, the axial velocity reduces rapidly. The physical interpretation is that an increase in *P*_*m*_ (Porosity) causes to produce more resistance to the fluid particles, and the magnetic dipole attracts the ferrite Fe_2_O_4_ (magnetite ferrite), MnZnFe_2_O_4_ (manganese zinc ferrite), and NiZnFe_2_O_4_ (Nickel zinc ferrite) nanoparticles which result in the enhancement of the viscosity of the nanofluid inside the nano boundary layer and as a result the axial velocity slow down. The highest velocity is observed for the C_2_H_6_O_2_ (ethylene glycol, when *φ* = 0) and H_2_O (water, when *φ* = 0), whereas the lowest axial velocity is observed for the Fe_2_O_4_—C_2_H_6_O_2_ (magnetite ferrite-ethylene glycol, when *φ* = 0.15) and Fe_2_O_4_—H_2_O (magnetite ferrite-water, when *φ* = 0.15) nanofluids as evident in Fig 7.

**Fig 8:**
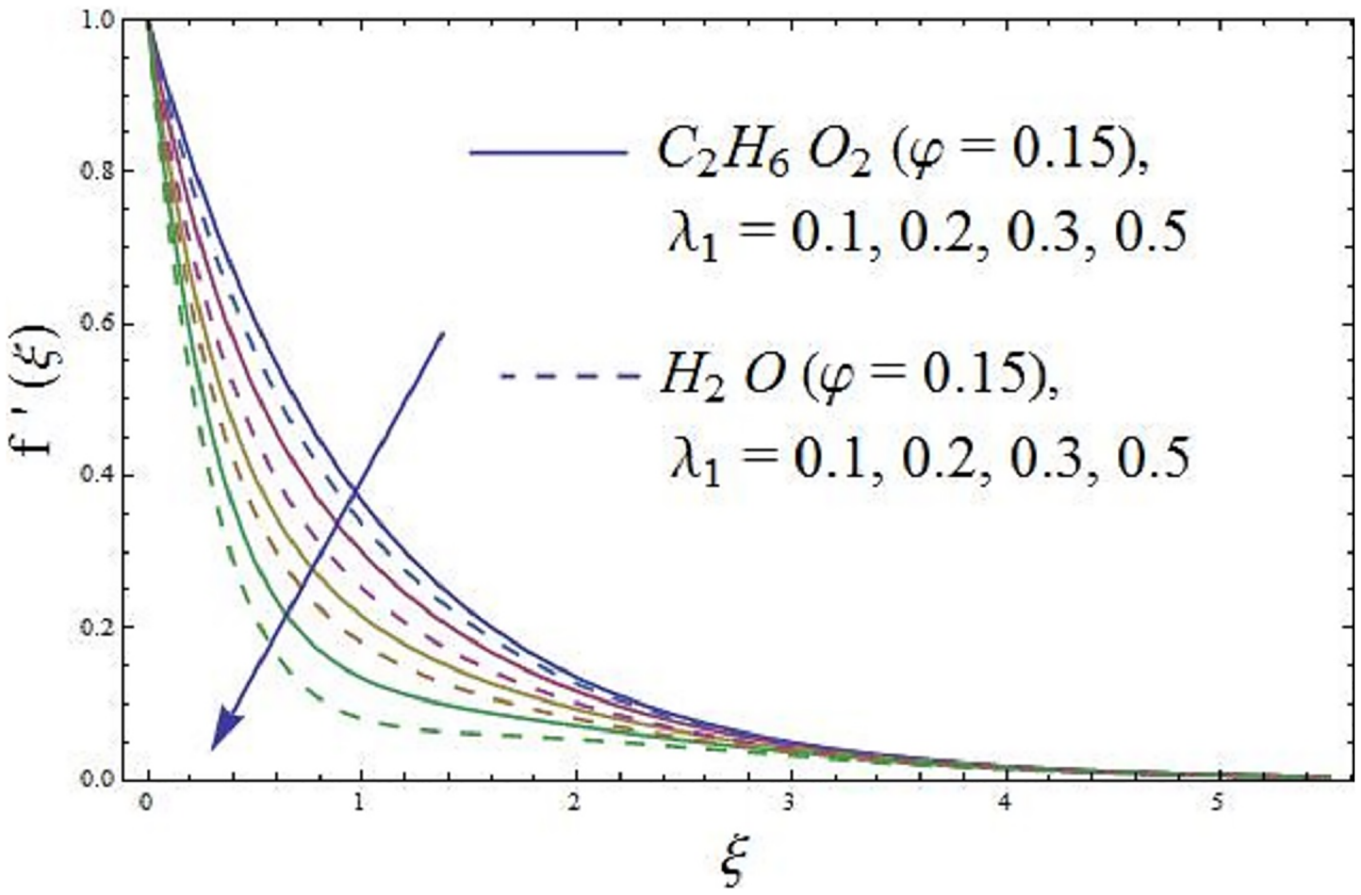
Effect of parameter λ_1_ (the conjugate parameter of Newtonian heating) on axial velocity.

**Fig 9:**
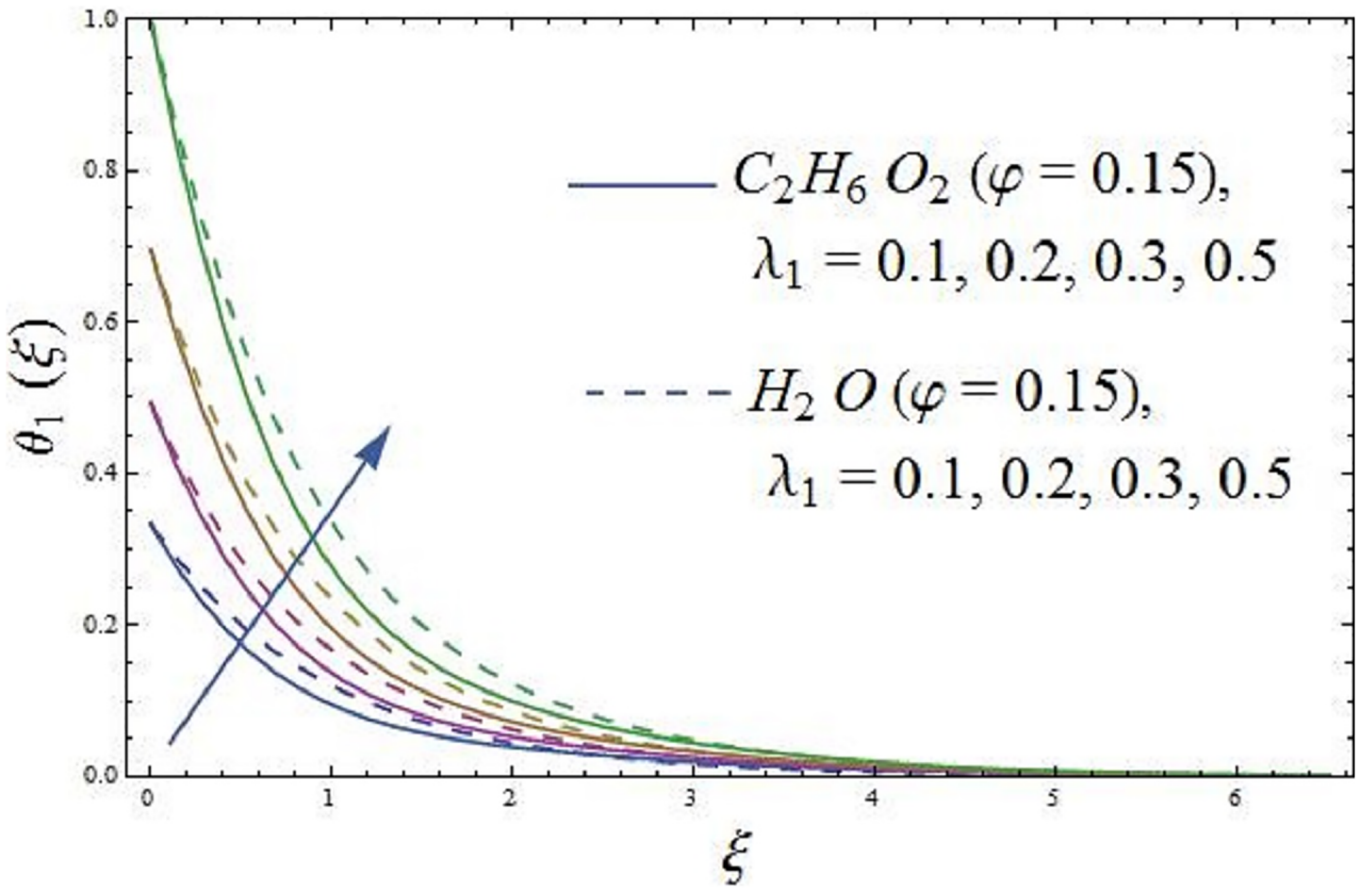
Effect of parameter λ_1_ (the conjugate parameter of Newtonian heating) on temperature field.

The influence of conjugate parameter λ_1_ of Newtonian heating on axial velocity and temperature profile are addressed in Figs 8 and 9. It is disclosed from Fig 8 that an increase in the λ_1_ (conjugate parameter) prompts change in the axial velocity of the ferromagnetic NiZnFe_2_O_4_—C_2_H_6_O_2_, NiZnFe_2_O_4_—H_2_O, MnZnFe_2_O_4_—C_2_H_6_O_2_, MnZnFe_2_O_4_—H_2_O, Fe_2_O_4_—C_2_H_6_O_2_, and Fe_2_O_4_—H_2_O nanofluids, the consequence indicate that the axial velocity and relative nano boundary layer are decreasing functions of λ_1_ (conjugate parameter) i.e., the axial velocity is reduces. It is inspected that the response velocity reduces with rise (increasing values of λ_1_) of an elastic force of the working fluid. The impacts of λ_1_ (conjugate parameter) on temperature field is characterized in Fig 9. It is revealed that an increase in λ_1_ (conjugate parameter) increases the heat transfer coefficient which improves the temperature of the ferromagnetic NiZnFe_2_O_4_—C_2_H_6_O_2_, NiZnFe_2_O_4_—H_2_O, MnZnFe_2_O_4_—C_2_H_6_O_2_, MnZnFe_2_O_4_—H_2_O, Fe_2_O_4_—C_2_H_6_O_2_, and Fe_2_O_4_—H_2_O nanofluids. Further, thermal boundary layer thickness increases. It is likewise noticed that temperature at the surface is higher for large values of λ_1_ (conjugate parameter).

**Fig 10:**
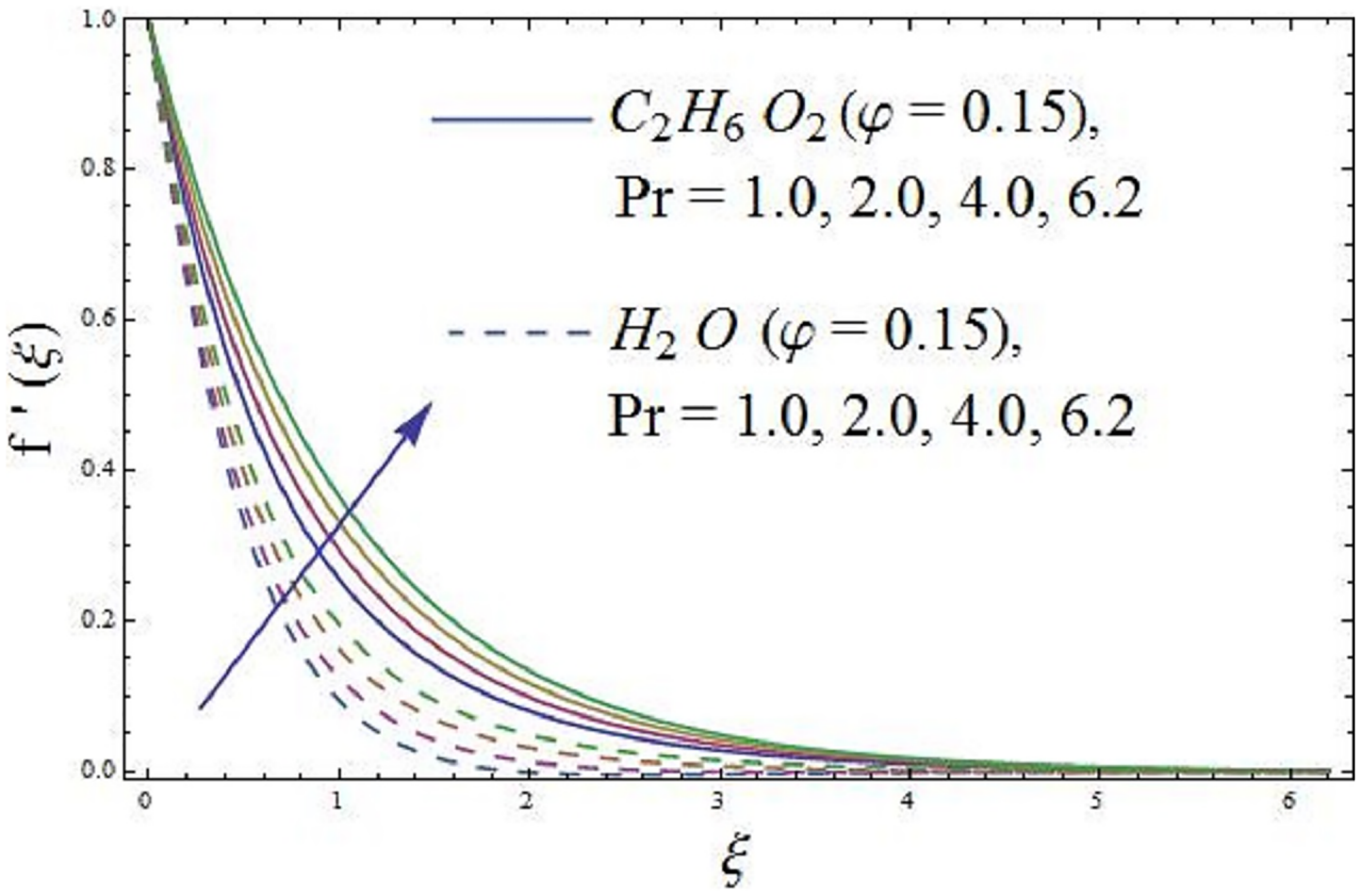
Impact of parameter Pr (Prandtl number) on axial velocity.

**Fig 11:**
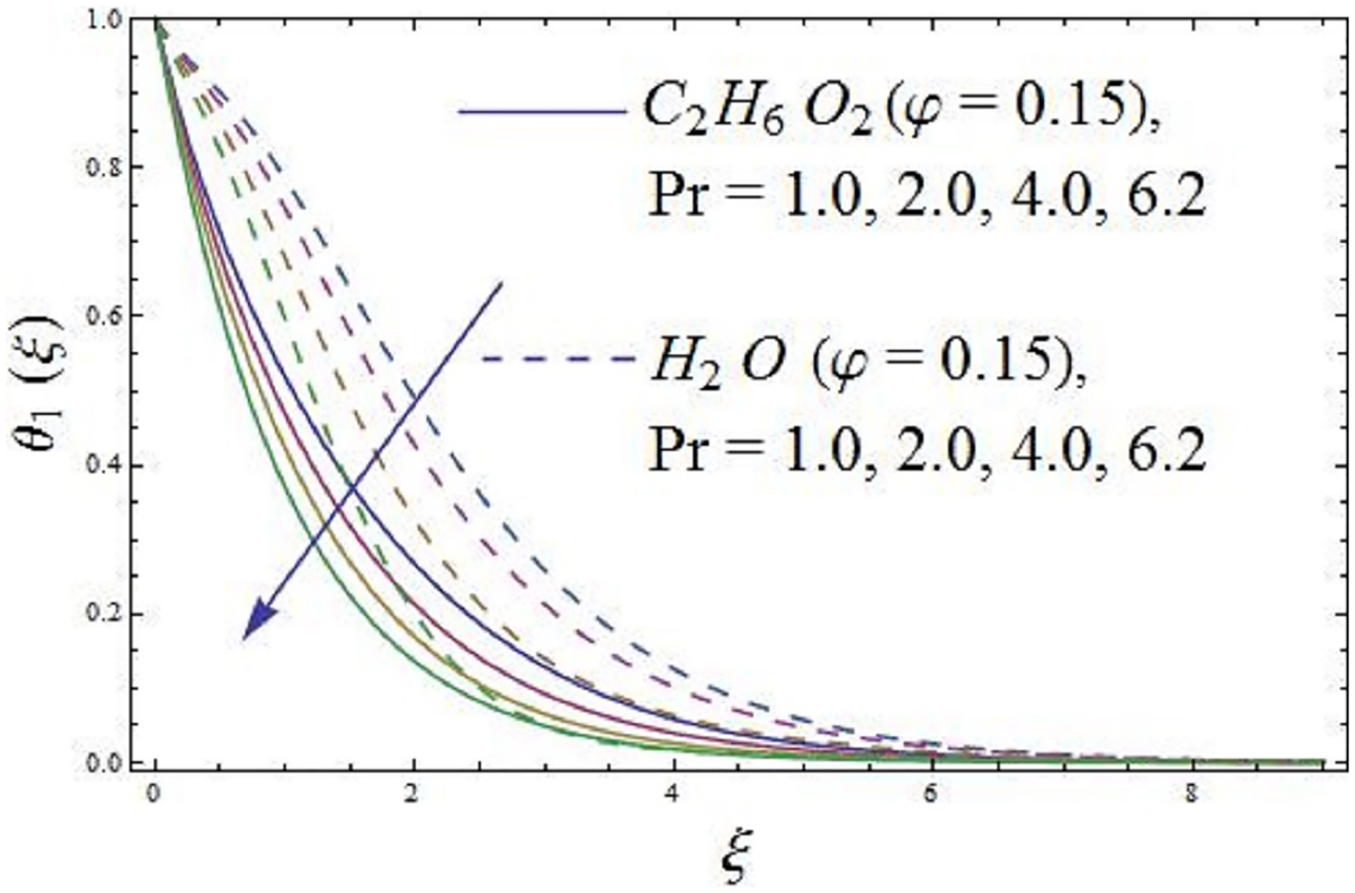
Impact of parameter Pr (Prandtl number) on temperature field.

The parameter Pr (Prandtl number) in the thermal energy equation effect their respective thermal boundary layer thickness. It is depicted in Fig 10 that higher values of parameter Pr (Prandtl number) enhances the axial velocity. Fig 11 exhibit the influence of parameter Pr (Prandtl number) temperature field of the ferromagnetic NiZnFe_2_O_4_—C_2_H_6_O_2_, NiZnFe_2_O_4_—H_2_O, MnZnFe_2_O_4_—C_2_H_6_O_2_, MnZnFe_2_O_4_—H_2_O, Fe_2_O_4_—C_2_H_6_O_2_, and Fe_2_O_4_—H_2_O nanofluids. It is investigated that the temperature field along thermal boundary layer thickness reduces for higher values of parameter Pr (Prandtl number) in presence of the magnetic dipole. A higher penetrating depth of temperature field is noticed at Pr = 1.0, as compared with Pr = 6.2, which results in the reduction of thermal diffusivity as parameter Pr (Prandtl number) rises. Reduction in thermal diffusivity leads to diffused heat away from the heated sheet and by the way, the temperature gradient at the surface is enhanced. This phenomenon decreases the ability of energy that decline the thickness of thermal boundary layer and enhance the axial velocity. The values assigned to remaining parameters are λ = 0.01, λ_1_ = 0.3, *β* = 1.2, Pr = 6.96, *P*_*m*_ = 0.5, and *γ* = 0.1.

### 5.1 Skin friction coefficient and local Nusselt number

**Fig 12:**
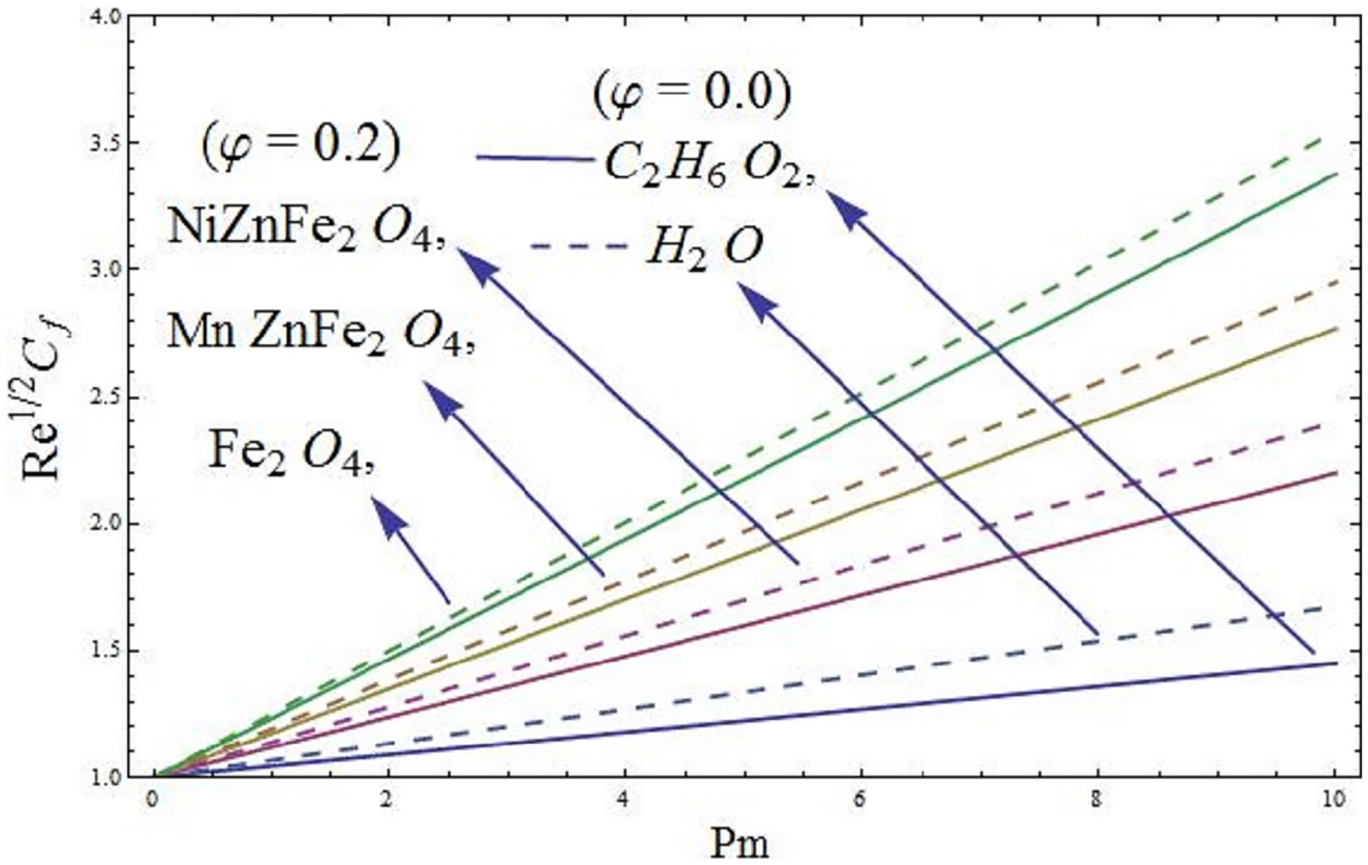
Wall shear stress versus *P*_*m*_.

**Fig 13:**
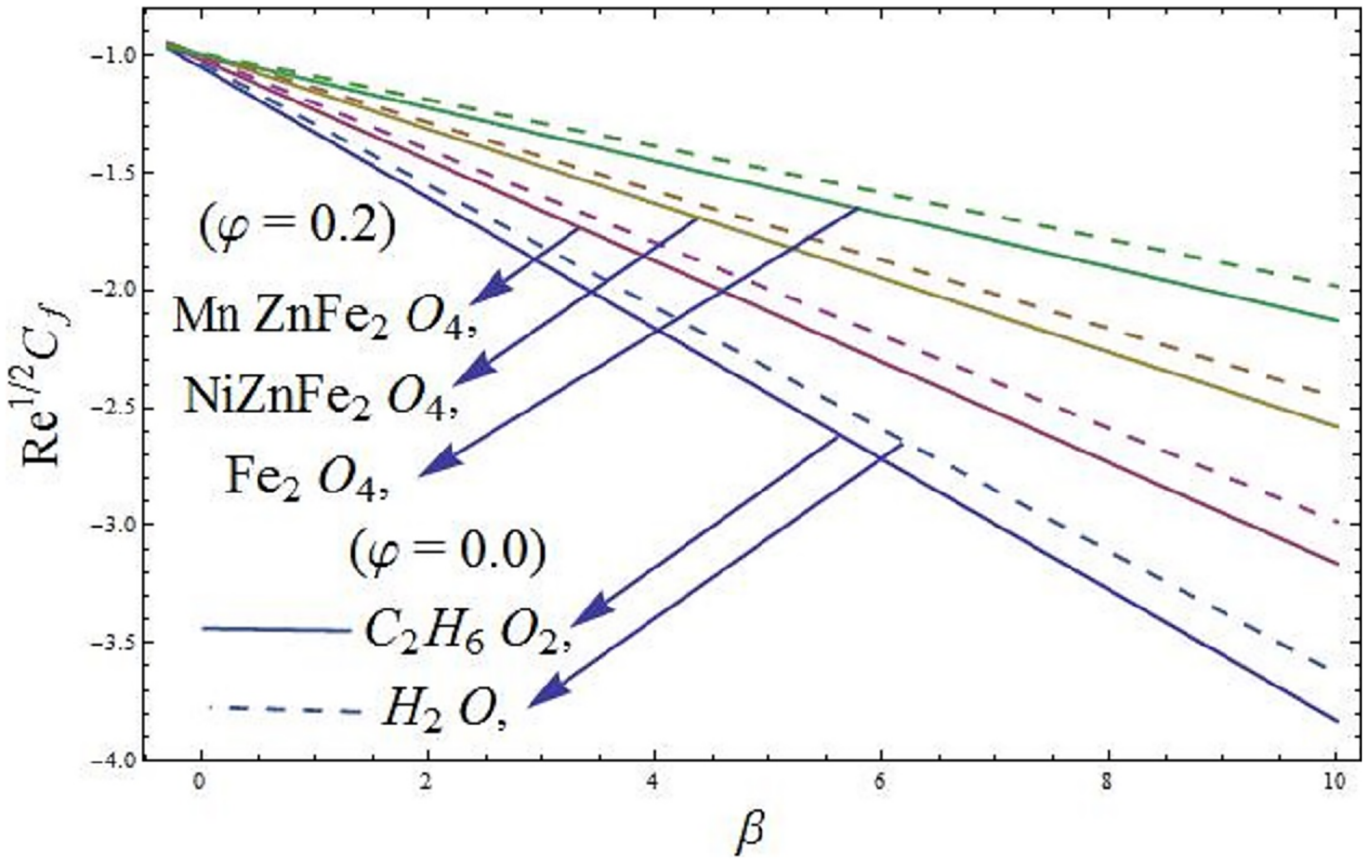
Wall shear stress versus *β*.

**Fig 14:**
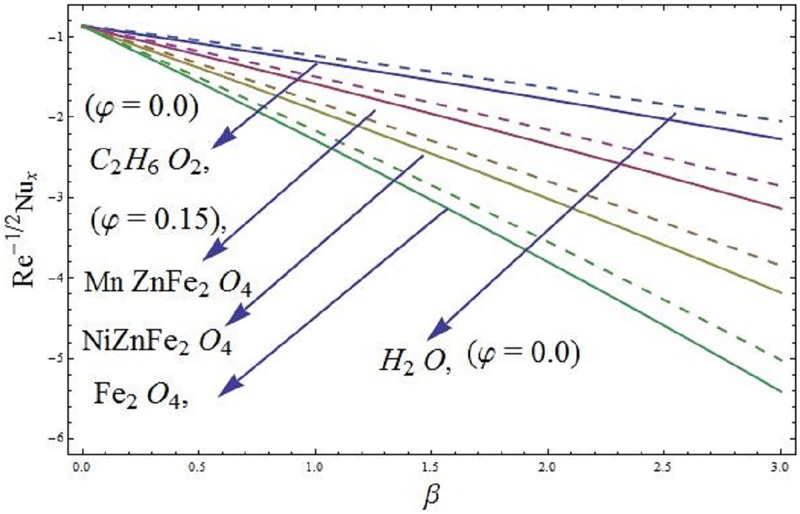
Heat transfer rate versus *β* (ferrohydrodynamic interaction parameter).

**Fig 15:**
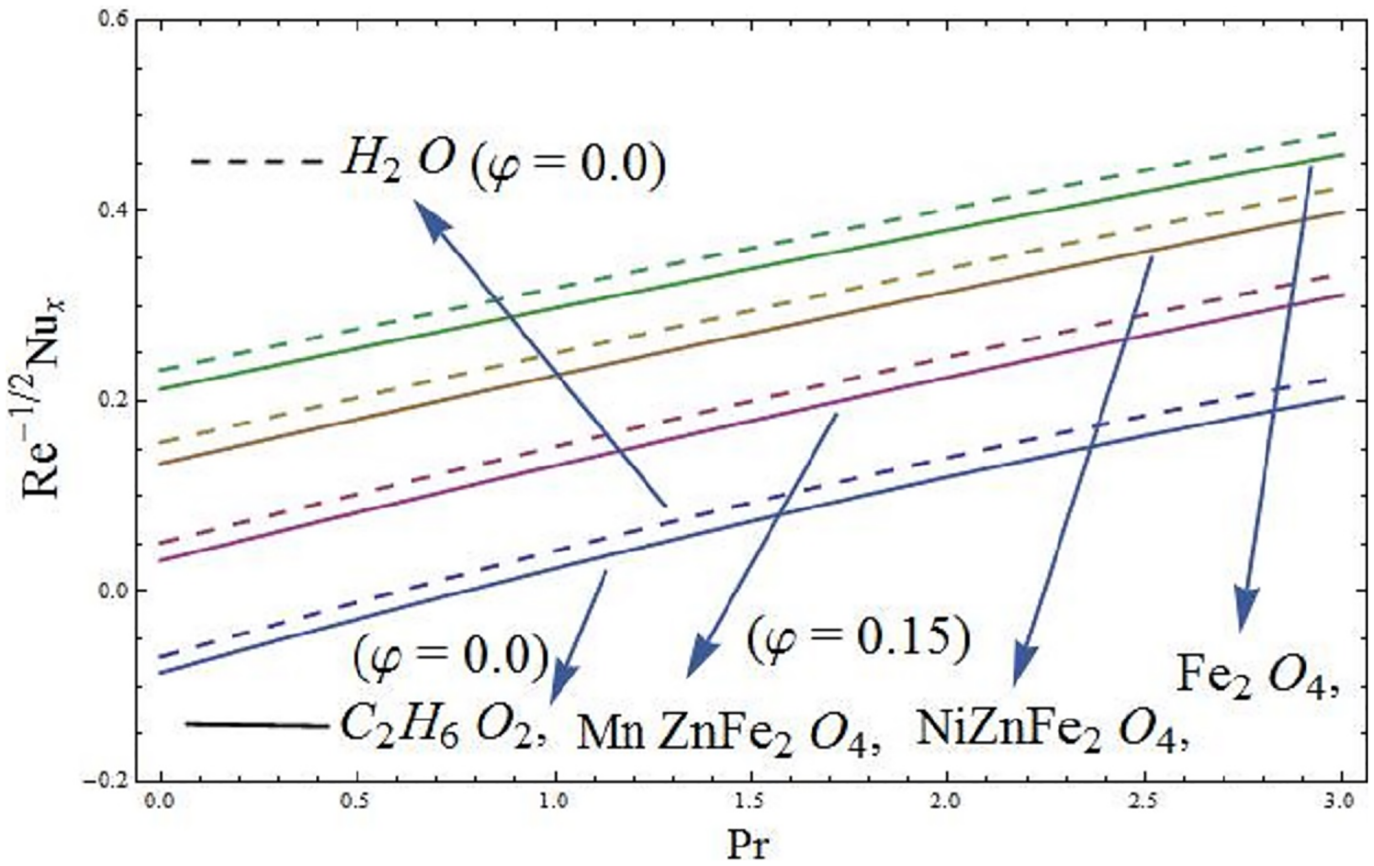
Heat transfer rate versus parameter Pr (Prandtl number).

The mathematical relations for skin friction coefficient and Nusselt number are given in Eqs 22 and 23. The influence of parameter *φ* (solid volume fraction of nanofluid) on wall shear stress of the ferromagnetic NiZnFe_2_O_4_—C_2_H_6_O_2_, NiZnFe_2_O_4_—H_2_O, MnZnFe_2_O_4_—C_2_H_6_O_2_, MnZnFe_2_O_4_—H_2_O, Fe_2_O_4_—C_2_H_6_O_2_, and Fe_2_O_4_—H_2_O nanofluids in presence of the magnetic dipole are evident in Fig 12. It is seen that the presence of water based ferrite nanoparticles reduces the wall shear stress as compared to the case when ethylene glycol-based ferrite nanoparticles are used. The skin friction coefficient is analyzed in the presence of magnetic dipole. Since we know that the magnetic dipole attracts the Fe_2_O_4_ (magnetite ferrite), NiZnFe_2_O_4_ (Nickel zinc ferrite) and MnZnFe_2_O_4_ (manganese zinc ferrite) nanoparticles which result in the enhancement of the viscosity of the nanofluid inside the boundary layer and yet the wall shear stress increases. The lowest wall shear stress is depicted for the C_2_H_6_O_2_ (ethylene glycol, when *φ* = 0) and H_2_O (water, when *φ* = 0) and the highest wall shear stress is observed for the Fe_2_O_4_—C_2_H_6_O_2_ (magnetite ferrite-ethylene glycol, when *φ* = 0.15) and Fe_2_O_4_—H_2_O (magnetite ferrite-water, when *φ* = 0.15) nanofluids as evident in Figs 12 and 13. Moreover, *φ* (solid volume fraction of nanofluid) on wall shear stress of the ferromagnetic NiZnFe_2_O_4_—C_2_H_6_O_2_, NiZnFe_2_O_4_—H_2_O, MnZnFe_2_O_4_—C_2_H_6_O_2_, MnZnFe_2_O_4_—H_2_O, Fe_2_O_4_—C_2_H_6_O_2_, and Fe_2_O_4_—H_2_O nanofluids in the presence of the magnetic dipole via heat transfer rate are analyzed in Figs 14 and 15. It is scrutinized from Fig 14 that the heat transfer rate reduces for ferromagnetic NiZnFe_2_O_4_—C_2_H_6_O_2_, NiZnFe_2_O_4_—H_2_O, MnZnFe_2_O_4_—C_2_H_6_O_2_, MnZnFe_2_O_4_—H_2_O, Fe_2_O_4_—C_2_H_6_O_2_, and Fe_2_O_4_—H_2_O nanofluids. The fast reduction in heat transfer rate is observed in presence of water based ferrite nanoparticles, instead, from Fig 15, it is evident that an increase in heat transfer rate is depicted for the respective ferromagnetic nanofluids. The values assigned to remaining parameters are λ = 0.01, λ_1_ = 0.3, *β* = 1.2, *P*_*m*_ = 0.5, Pr = 6.96, and *γ* = 0.1.

#### Concluding remarks

The purpose of the article exhibit theoretically the practicability of the concept of ferromagnetic nanofluids with Fe_2_O_4_ (magnetite ferrite), NiZnFe_2_O_4_ (Nickel zinc ferrite), and MnZnFe_2_O_4_ (manganese zinc ferrite) as ferrites nanoparticles and H_2_O (water) and C_2_H_6_O_2_ (ethylene glycol) as base fluid. The heat transport phenomenon is depicted in the resulting ferromagnetic nanofluids. The boundary value problem is solved numerically and analytically with the help of BVPh2—mid point method and optimal homotopy analysis method respectively. The main points of the analysis are:

▸ An increase in *φ* (solid volume fraction of nanofluid) results in the reduction of axial velocity and enhances the temperature field.
▸ The existence of parameters *P*_*m*_ (Porosity) in the presence of Fe_2_O_4_ (magnetite ferrite), NiZnFe_2_O_4_ (Nickel zinc ferrite) and MnZnFe_2_O_4_ (manganese zinc ferrite) nanoparticles in a viscous carrier ferromagnetic nanofluid slow down the axial velocity.
▸ Axial velocity reduces and the temperature field enhances fastly for increasing values of *β* (ferromagnetic interaction) when magnetic dipole is present.
▸ The axial velocity and relative nano boundary layer of the ferromagnetic NiZnFe_2_O_4_—C_2_H_6_O_2_, NiZnFe_2_O_4_—H_2_O, MnZnFe_2_O_4_—C_2_H_6_O_2_, MnZnFe_2_O_4_—H_2_O, Fe_2_O_4_—C_2_H_6_O_2_, and Fe_2_O_4_—H_2_O nanofluids are decreasing functions of λ_1_ (conjugate parameter) and increasing function of temperature profile.
▸ Prandtl number results in the rapid depletion of the temperature field while enhancement in axial velocity in presence of magnetic dipole.
▸ The wall shear stress of the ferromagnetic NiZnFe_2_O_4_—C_2_H_6_O_2_, NiZnFe_2_O_4_—H_2_O, MnZnFe_2_O_4_—C_2_H_6_O_2_, MnZnFe_2_O_4_—H_2_O, Fe_2_O_4_—C_2_H_6_O_2_, and Fe_2_O_4_—H_2_O nanofluids enhances with parameters *β* (ferromagnetic interaction) and *P*_*m*_ (porosity).
▸ The fast reduction in heat transfer rate is observed in presence of magnetic dipole.

#### Future work

The present study can be investigated by incorporating convective boundary conditions, Newtonian heating, Darcy and non-Darcy porous media, Cattaneo-Christove heat and mass fluxes, variable thermal conductivity, variable mass diffusivity, Corban nanotubes etc. The features of ferrite nanoparticles can be disclosed in viscous fluids with various geometries. It will be considered in the near future with different kinds of fluid models. It is hoped that present study serves as a stimulus for drug delivery in biomedical processes.
